# A perspective on digital health platform design and its implementation at national level

**DOI:** 10.3389/fdgth.2024.1260855

**Published:** 2024-04-11

**Authors:** Manisha Mantri, Gaur Sunder, Sanjay Kadam, Aditya Abhyankar

**Affiliations:** ^1^ HPC-Medical & Bioinformatics Application Group, Centre for Development of Advanced Computing (C-DAC), Pune, India; ^2^ National Supercomputing Mission, Centre for Development of Advanced Computing (C-DAC), Pune, India; ^3^ Department of Technology, Savitribai Phule Pune University (SPPU), Pune, India

**Keywords:** digital health platform, WHO, ITU, interoperability, standards, India, NDHM

## Abstract

Accessible and affordable health services and products including medicines, vaccines, and public health are an important health agenda of all countries. It is well understood that without digital health technologies, countries will face difficulties in tackling the needs and demands of their population. Global agencies including the World Health Organization (WHO), United Nations (UN), International Telecommunication Union (ITU), etc. have been instrumental in providing various tools, and guidance through digital health strategies in improving health and digital health maturity of the countries. The Digital Health Platform Handbook (DHPH) is a toolkit published by WHO and ITU to help countries create and implement a digital health platform (DHP) to serve as the underlying infrastructure for an interoperable and integrated national digital health system. We apply the foundational principles of DHPH and provide a perspective of DHP components in a layered, enterprise architecture of a digital health infrastructure. India has rolled out the blueprint of its National Digital Health Mission (NDHM) to address the emerging needs for digitization of healthcare in the country. In this paper, we also illustrate the design and implementation of WHO-ITU DHP components at the national level by exploring India's digital health mission implementation utilizing various digital public goods to build a digital health ecosystem in the country.

## 1 Introduction

Technology usage and innovation in healthcare are continuously improving the delivery and quality of health services by aiding in diagnosis and treatment in preventive, curative, and palliative care. Accessible and affordable health services and products including medicines, vaccines, and public health services are an important health agenda of all countries and the World Health Organization (WHO). Health technology is one of the key drivers for achieving sustainable development goals for health. Technology has played an extremely important role during the COVID-19 pandemic in reporting, analyzing, and planning health interventions during the outbreak along with the distribution and rollout of vaccines. It is well understood that without technological health interventions, countries will face difficulties in tackling the needs and demands of their population.

Health is the third key goal of the United Nations (UN) Sustainable Development Goals (SDGs). Many countries have evolved eHealth strategies to improve health services in their countries. To harmonize and support the use of technology in health across the world in a standardized and interoperable manner, WHO along with the International Telecommunication Union (ITU) has, over time, announced different eHealth strategies aligned with the UN SDGs. The National eHealth Strategy Toolkit by WHO and ITU provides a set of basic components and processes to focus upon while developing a national eHealth strategy (1). It provides a broader vision of health system development and enables countries to shape the development of a national eHealth framework. The toolkit works as a guide for countries to develop their eHealth strategies and a benchmark for assessment of the implementation progress (2–7). Liaw et al. prepared a list of indicators to describe a country's digital health profiles along with the digital health maturity assessment tool that uses criteria co-developed with country stakeholders for Pacific Island Countries referring to the Global Digital Health Index (GDHI) and MEASURE (Monitoring and Evaluation to Assess and Use Results) Evaluation and Health Data Collaborative Toolkits and Maturity Models (8–10). Further, in 2020, the WHO developed a Global Strategy for Digital Health for 2020–2025. The vision of the strategy was to improve health for everyone, everywhere by accelerating the development and adoption of sustainable person-centric digital health solutions to prevent, detect and respond to epidemics; developing infrastructure and applications that enable countries to use data to promote health and wellbeing, and to achieve the health-related UN SDGs (11). It provides a comprehensive definition of digital health as “the field of knowledge and practice associated with any aspect of adopting digital technologies to improve health, from inception to operation,”. United Nations through SDG 3.8.2 have set universal healthcare coverage (UHC) as a target, to be achieved by 2030 (12). Only the appropriate use of digital technologies can accelerate the development of sustainable health systems through digital health initiatives guided by a robust national digital health strategy (13). The ability to exchange and use information between different systems (interoperability) is a fundamental requirement to accomplish healthcare goals. With fragmented, incomplete, and isolated health systems, a lack of interoperability would lead to the loss of continuity of care. Developing countries facing a lack of adequate infrastructure have the fundamental need to develop nationwide e-health agendas to achieve sustainable implementations (14).

While WHO's Global Strategy on Digital Health outlines the overarching vision, core principles, and strategic objectives that should guide national digital health initiatives, there is a definite possibility of applying different methodologies and design principles for building a national health system. The Digital Health Platform Handbook (DHPH) has been announced by WHO and ITU as a toolkit to help countries create and implement a digital health platform (DHP) to serve as the underlying infrastructure for an interoperable and integrated national digital health system (15). DHPH is more implementation and interoperability-focused and complements the top-level vision of global strategy by providing a detailed walkthrough of the design and implementation of the digital health platform for countries (16). This paper provides a perspective of the high-level blueprint of the DHP, and its common components, and compares it with the rolled-out blueprint and plan of the National Digital Health Mission in India.

## 2 Digital health platform (DHP)

WHO and ITU define a digital health platform as a common digital health information infrastructure (“infostructure”) having an integrated set of common and reusable components that external digital health applications and systems can use to deliver digital health services in a standardized, interoperable, and integrated manner (16). The broader goal is to simplify the information exchange within the health sector, promote re-usability, reduce the complexity of implementation, and enable seamless healthcare service delivery. The DHPH envisages three major stages i.e., context analysis, design, and implementation in developing a DHP with multiple individual tasks.

The context analysis stage requires identifying the business process requirements and improvements. Prioritizing and mapping these requirements based on the survey of the current health system, healthcare actors, and existing digital health assets through literature review and stakeholder interviews. The DHP design stage involves defining clear, concrete, and concise DHP principles, outlining the enterprise architecture, identifying DHP components based on the business processes, and identifying and adopting appropriate standards. To foster interoperability and adoption, a few of the recommended DHP design principles referred to in the handbook from Open Group Architecture Principles include; the use of APIs, collaborative decision-making, use of open standards, promoting open-source development for wide adoption, re-usability, and innovation, data quality, and integrity, etc. A summary of the common DHP components required for any national/regional implementation is discussed further in the sub-section. A DHP implementation approach could be either a ground-up approach—design before you build, or a hub approach—build while you design following different models of implementations i.e., centralized, decentralized, or hybrid. The suitability of the model depends upon the identified business processes and priorities, infrastructure, and data policies and practices (17). The handbook emphasized the importance of establishing the governance framework for DHP implementation and institutionalizing it with the designated eHealth entity responsible for its execution and maintenance. The handbook also provides various instances of context analysis, design, and implementation of infrastructure with inline vision by various countries and regions.

### 2.1 Common DHP components

DHP components are services/protocols, internal to the digital health platform, that allow external digital health applications and systems to provide and access information. The DHP components are expected to be—sharable and reusable across the platform and external systems; use-case independent to be applicable across different business processes such as maternal health programs, immunization programs, elderly care, HIV treatment, etc. as required by various SDG goals, and always scalable to cater to the dynamic and futuristic requirements.

**Figure 1:**
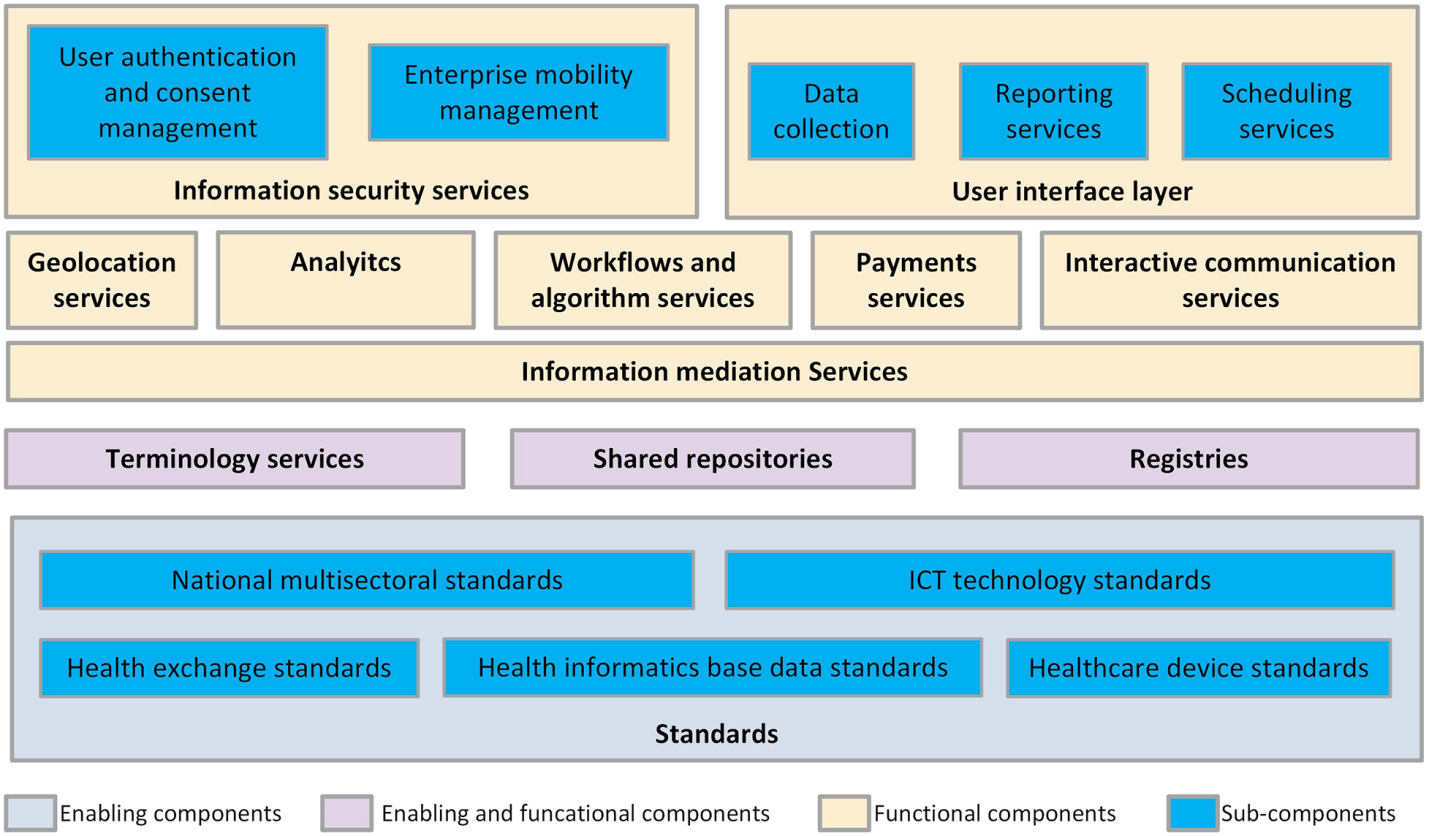
WHO-ITU common DHP components (16).

Figure 1 provides the stack of the DHP components along with the various standards. The DHPH envisages two categories of components; enabling components and functional components. In our view, apart from the common components discussed in the handbook, the standards required for building the interoperable components should also be considered as the enabling components for a DHP. The handbook provides various characteristics and examples of these components.

### 2.2 National digital health mission implementation in India

The Ministry of Health & Family Welfare (MoHFW), Govt. of India released the National Digital Health Blueprint (NDHB) in April 2019 following the visions of National Health Policy 2017 (18, 19). The document provides a plan to achieve the digitalization of health records across the country; the creation of unique digital health IDs; building and maintaining registries of healthcare providers, patients, important diseases, and drugs; linking health records; payment gateways; and provision standards and regulations within the operating framework regarding data management and security. The blueprint also recommends the mandate of the National Digital Health Mission (NDHM) for designing, developing, and realizing universal technology building blocks useful for the implementation of the mission.

NDHM, renamed now as Ayushman Bharat Digital Mission (ABDM), is guided by the NDHB, and follows the National Health Stack 2018 document released by the National Institute for Transforming India- NITI Aayog, which is an apex public policy think-tank of the Government of India (20). The NDHB adopts a layered framework for digital health infrastructure, various building blocks, standards, policies, etc. The ABDM vision is to—*create a national digital health ecosystem that supports universal health coverage in an efficient, accessible, inclusive, affordable, timely, and safe manner, that provides a wide range of data, information, and infrastructure services, duly leveraging open, interoperable, standards-based digital systems, and ensures the security, confidentiality, and privacy of health-related personal information* (21). ABDM has stated business processes that aim to develop the backbone network to support the integrated digital health infrastructure of the country with the adoption of specific policy and technology principles such as building a single source of truth (registries), adopting federated architecture, adopting India Enterprise Architecture Framework (IndEA), use of open standards and open-source software, inclusivity, wellness-focused implementation, etc. (21).

**Figure 2:**
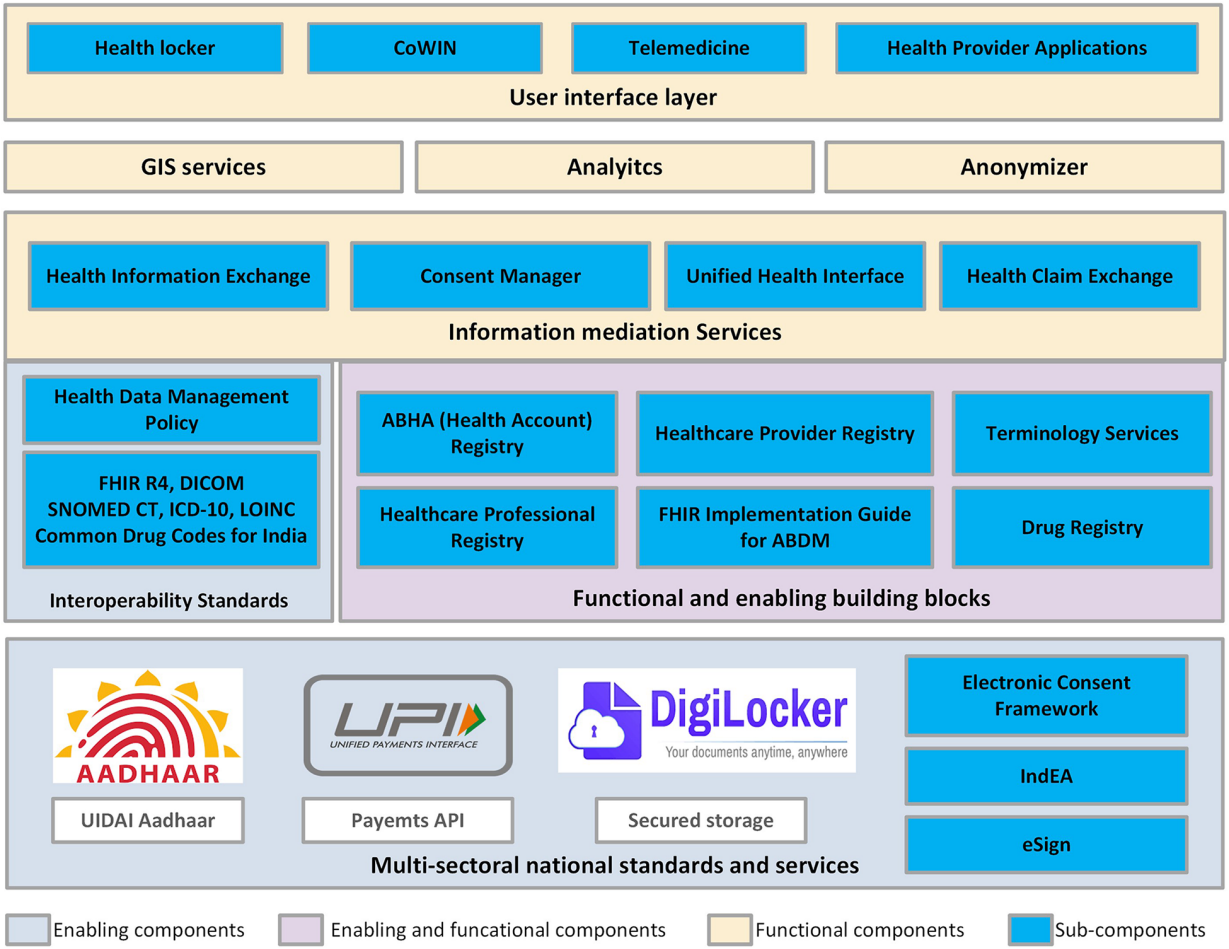
ABDM building blocks.

The various building blocks identified and implemented in ABDM (adapted from ABDM Architecture published by NHA) are depicted in Figure 2.

Among the multi-sectoral national standards and services, Aadhaar [“Foundation” in English] is a biometric-based unique identity system provided by the Unique Identification Authority of India (UIDAI. It offers unique Aadhaar IDs to the citizens that can be used by any e-governance system to verify the identity of a person for her any digital interactions across local regions as well as states (22). While Aadhaar is not mandatory for creating ABDM Unique Health ID or Ayushman Bharat Health Account (ABHA), it can be used or linked for various purposes. ABDM uses only ABHA to track various artifacts of an individual in the system.

The Unified Payment Interface (UPI), created by the National Payment Corporation of India (NPCI) is a next-generation mobile-based payment system that enables round-the-clock, real-time bank payments. UPI is designed to promote digital payments in India (23). It leverages the high teledensity in India by making the mobile phone a primary payment device for both consumers and merchants and universalizing digital payments in the country (24). With the successful implementation, the UPI model has been adopted in many e-governance initiatives as well as in ABDM. There was a significant increase in the usage of digital payment using UPI in healthcare, pharmaceutical, and insurance during and after the COVID-19 pandemic (25).

The DigiLocker is an initiative of the Ministry of Electronics & IT (MeitY) under its Digital India programme. It provides a mobile application (a digital document wallet) that provides access to authentic digital documents to the citizen. It supports the storage, sharing, and verification of documents & certificates including Aadhaar and other proofs of identity and address, educational certificates, and recently the COVID-19 vaccine certificate, etc. (26, 27). The architecture of DigiLocker is further adopted for developing the Health Locker under the ABDM.

The country has adopted a national consent management technical framework for consent-taking in all the e-governance programmes and initiatives. ABDM Consent Manager building block also leverages the same consent management framework along with standard *ISO/TS 17975:2015 Health Informatics - Principles and data requirements for consent in the collection, Use or Disclosure of personal health information*.

The online electronic signature service commonly referred to as eSign services of Govt. of India is one of the initiatives of moving to fully paperless citizen services. The services licensed by the Controller of Certifying Authorities (CCA) can be integrated into any service delivery applications to facilitate eSign for digitally signing documents (28). This infrastructure has been further planned for usage in ABDM for verification of health care providers and enabling them to sign the health records digitally for authentication and non-repudiation.

The standards have been followed by applying a minimalistic approach to data sharing i.e., mandate only the essential and minimum health information for supporting continuity of care. The various health data standards adopted include HL7 FHIR as structural data standard, DICOM for medical image representation and sharing, SNOMED CT as clinical terminology standard, ICD-10 for reporting and classification, LOINC for laboratory observations and measurements, and Common Drug Codes for India developed by the National Resource Centre for EHR Standards (NRCeS) (29, 30). ABDM has a Health Data Management Policy that provides guidance and a framework for the secure processing of health records of individuals under ABDM (31).

The enabling and functional building blocks include the digital registries and FHIR Implementation Guide for ABDM, which serve as a single source of truth for all the components (32–35). The Terminology service is planned and the Drug Registry which uses the Common Drug Codes for India is piloted and not yet implemented in production (30, 36).

Under information and mediation services, the Health Information Exchange (HIE) works as a Gateway for all the Health Information Provider (HIP) and Health Information User (HIU) entities for the exchange of health data. The HIE stores the indexes of the health records for searching and forwarding the requests based on the patient consent through the consent manager. Unified Health Interface (UHI) and Health Claim Exchange (HCX) are separate gateways implemented using an open protocol. UHI enables health service discovery and delivery by utilizing the enabling and functional building blocks of ABDM. UHI ensures interoperability in health services offered by a variety of participating providers from any application (37). The HCX aims to automate the health claim-related information exchange between payers, providers, beneficiaries, and other relevant entities. HCX gateway is currently in the initial stage of the implementation while UHI is not mandated. Their progressive adoption timelines have been set by NHA to balance the adoption burden amongst implementers. The GIS Service, Analytics, and Anonymizer are currently at the conceptual stage as the implementation of these services requires sufficient data and data-sharing policies. Currently, the DHP only refers to a broad metadata of the health records which is required for identification and indexing of the health records in HIE.

Several reference applications are developed to demonstrate the capabilities of these building blocks including PHR application, Health Locker, ABHA application, WebEMR for healthcare providers, etc. The other functional services such as health GIS, anonymizer service, health analytics service, etc. are also developed and planned as envisaged in NDHB.

## 3 Discussion

The ABDM has been rolled out in the country with its major building blocks and services including Patient, Provider, and Facility registries, Health Information Exchange, and FHIR based health records from 2021. The mission is in the middle of its implementation and has enabled the linking of over 300 million health records across various hospitals in the country, thus far (38, 39). Despite being designed earlier, the ABDM building blocks and the architecture largely follow DHPH guidelines. The ABDM implementation is focused on wellness and continuity of care. The architecture is based on the IndEA framework, which is based on the most comprehensive, widely used, and accessible enterprise architectures, The Open Group Architecture Framework (TOGAF), a standard of the Open Group (40). DHPH provides guidelines to define the DHP design principles such as privacy and security, use of APIs, collaboration, open standards, open source, usability, Scalability, data custodianship, and policy adherence. The ABDM design is based on the major principles divided clearly into Technology Principles including a single source of truth; federated architecture; open APIs; standards and interoperability; and adherence to national IndEA framework; and the Policy Principles including privacy and security by design; user inclusivity; wellness centric; and voluntary participation. ABDM implementation is also governed by a strong framework through the establishment of NHA, stakeholder engagement via regular consultations for implementation of registries and ABDM building blocks, development of the network infrastructures across the country, and reusable nationwide services like Aadhar, DigiLocker, eSign, etc.

ABDM uses a decentralized architecture for data management, where the data resides at the healthcare facilities, and patient consent-based access is provided to the requesting healthcare provider through HIE APIs. The decentralized architecture has its challenges as they are costly to implement, always requires the availability of every participant, is resource-intensive to maintain, and is not scalable to accommodate changes, especially across the entire ecosystem of healthcare. They also pose challenges to trust in the network and hence may require exploring technologies such as blockchain (41, 42). It should also be noted that DHP implementation may vary based on the country's priorities, policy environment, and available resources. Another good early example of a large-scale, nationwide DHP design is developed by Canada Health Infoway, called EHRS Blueprint. This blueprint depicts the information system architecture for various healthcare applications using shared infrastructure (43).

The current structure of the health records uses FHIR R4 Documents format. The health records are designed to support historical data i.e., PDFs and scans, structured text data, as well as terminology encoded data using SNOMED CT, ICD, and LOINC (44). This approach has helped in the quick adoption of the standard structure. However, most of the hospitals and health systems are currently supporting only the first format of data. Such an approach further requires technologies like OCR, AI-ML, etc., with high precision for machine processability of health records. Real interoperability can only be achieved when all the health systems in the ecosystem adopt the third form of data sharing. This requires the components such as the terminology services to be implemented and made available for data capture and validation.

The ABDM implementation aims to transform the health sector into an open, collaborative, interconnected ecosystem structured around an architecture of integrated digital services, the fact that participation in ABDM is voluntary for the healthcare providers as well as patients, poses challenges to its nationwide adoption. The availability of infrastructure and critical health data also lays the requirements for ensuring strict data safety. The data management policies and consent framework help in advocating the safety and legitimate use, storage, and access of data, however a strong data protection bill as part of the legislation is necessary to safeguard the patient's privacy and rights on data usage. Although regulations as powerful, countries often try to balance the needed regulations. They take a long time to implement and are hard to change over time (45, 46). On the other hand, the flexible nature of policies is often better suited to the fast-changing field of digital technology. Hence with the constantly changing technology, in digital health, a balance of regulations and policies should be sought. The regulations should be implemented where patients' privacy and data protection could be compromised. Policies should be developed on the mechanisms and methods of handling data and processes. For example, for data protection, there is a Digital Personal Data Protection Act implemented in India (47) while data storage and handling guidelines are provided through a data management policy by NHA which will be updated from time to time.

There are strong requirements of compliance and certification for the integration of interoperability standards in ABDM for onboarding healthcare software. The platform requires different levels of compliance in terms of meeting the Minimum Viable Product (MVP), milestones and further the health systems go through a voluntary functional evaluation to ensure its meaningful use to improve the quality of healthcare processes and services (48). The research shows even after going through the mandatory certification process, due to different design choices, feature offerings, and computerization focus, the in-field experience of usability of the health systems may be completely different and may affect the performance of healthcare facilities (49, 50). As a step towards institutionalizing the adoption of digital health platforms, policymakers should consider strengthening the certification scheme to minimize such functional differences or enable mechanisms to differentiate the systems based on their functions.

Establishing the governance framework and institutionalization are the next key steps after the DHP implementation. ABDM currently stands at the last step and there are multiple activities undertaken for institutionalization such as the implementation of the Digital Health Incentive Scheme (DHIS) (51), demonstration of smart digital hospitals at Primary Health Centers (PHCs) through PPP models called microsite implementations, developing multiple use cases under ABDM to showcase accessibility and delivery of healthcare services to the stakeholders, etc. Despite the rapid progress of the mission, there is a need for larger participation from the public as well as private healthcare providers. The mission should also develop a road map for its maturity through capacity building, monitoring, and periodic impact assessment (38). Success in healthcare innovation and transformation is possible only if the health workforce is ready to adopt the technology. The common barriers to the adoption of digital health technologies in the healthcare workforce in countries including India are health providers' skills, knowledge, confidence, and fears of technological separation affecting healthcare service delivery (52, 53). While the solutions are onboarded, the policies, initiatives, and programs for digital health workforce development are particularly important for the widespread adoption of digital health applications (54). Promoting digital health translation through investment in capacity-building programs at hospitals, medical colleges, and universities along with demonstrating the key benefits of the digital health ecosystem in practice is crucial for the successful adoption of the mission.

Effective utilization of healthcare technology also requires a certain level of digital literacy in patients, which is unevenly distributed across the country. Presently, although India has the highest rates of smartphone usage globally, gender and age disparities and the digital literacy divide still exist (55). Addressing the digital divide through digital literacy policies and implementation in a collaborative approach involving public, private, and non-profit entities is crucial for the successful implementation of the mission.

This paper presents an approach to implementing DHP components in a layered, reusable, and holistic manner. The WHO-ITU Common DHP Components, Figure 1 can be used as a reference architecture for evaluating and designing a digital health enterprise architecture at any scale. This is one of the ways of depicting the WHO components and there might be several other depictions possible. The layered enterprise architecture of DHP enables the reusability of the technology components while digital health standards such as HL7 FHIR, DICOM, SNOMED CT, ICD, and LOINC enable the reusability of data in a federated digital health ecosystem. A well-defined policy and technology principles with appropriate governance in place can help in providing a clear vision for implementing a DHP. A step-by-step approach to the adoption of digital health services is a feasible mechanism to avoid the burden of implementation. Countries promote incentive schemes for digital health adoption which many times pose challenges to the data quality. This necessitates the use and implementation of Information Quality Frameworks which is currently missing (56). While promoting adoption, appropriate majors must be taken to ensure the completeness and correctness of health records through certification schemes, MVP guidelines, IQ frameworks, etc. so that the data can be used for secondary healthcare purposes. This work focuses mainly on the design and implementation of a DHP, and this can further be extended by defining a common framework for governance and institutionalization referring to DHPH guidelines.

## Data Availability

The original contributions presented in the study are included in the article/Supplementary Material, further inquiries can be directed to the corresponding author.
